# Modulation of the Differentiation of Dental Pulp Stem Cells by Different Concentrations of β-Glycerophosphate 

**DOI:** 10.3390/molecules17021219

**Published:** 2012-01-31

**Authors:** Mingyue Liu, Yao Sun, Yang Liu, Mengtong Yuan, Zhihui Zhang, Weiping Hu

**Affiliations:** 1 Department of Prosthodontics, the 2nd Affiliated Hospital of Harbin Medical University, Harbin, Heilongjiang 150086, China; Email: drliumingyue@163.com (M.L.); 554330009@qq.com (M.Y.); 2 Institute of Hard Tissue Development and Regeneration, the 2nd Affiliated Hospital of Harbin Medical University, Harbin, Heilongjiang 150086, China; Email: sunyao919@126.com; 3 Department of Stomatology, Mianyang Central Hospital, Mianyang, Sichuan 150086, China; Email: 65583911@qq.com; 4 School of Stomatology, Peking University, Beijing, 100081, China; Email: zhangzhihui8411@sina.com

**Keywords:** MEPE, DSP, β-glycerophosphate, dental pulp stem cells, odontoblast

## Abstract

Dentinogenesis is a necessary prerequisite for dental tissue engineering. One of the steps for dentinogenesis is to obtain large quantities of highly purified odontoblasts. Therefore, we have undertaken an experiment applying different concentrations of β-glycerophosphate (β-GP) to induce the differentiation of dental pulp stem cells (DPSCs) in a long-term 28-day culture. In the meanwhile, we have studied the time- and maturation-dependent expression of matrix extracellular phosphoglycoprotein (MEPE) and that of the odontoblast-like marker-dentin sialoprotein (DSP), in order to investigate an optimized mineralized condition. Western blot results revealed that the expression of DSP became lower when accompanied by the increase of the β-GP concentration, and there was also an influence on MEPE expression when different concentrations of β-GP were applied. Meanwhile, the mineralized groups had an inhibitory function on the expression of MEPE as compared with the control group. Above all, all experimental groups successfully generated mineralized nodules by Alizarin Red S and the 5 mM β-GP group formed more mineralized nodules quantitated using the CPC extraction method. In conclusion, there is a significant modulation of the β-GP during the differentiation of the DPSCs. The degree of odontoblast differentiation is β-glycerophosphate concentration dependent. A low concentration of β-GP (5 mM) has been shown to be the optimal concentration for stimulating the maturation of the DPSCs. Moreover, MEPE accompanied with DSP clearly demonstrates the degree of the differentiation.

## 1. Introduction

Human dental pulp stem cells (DPSCs), like bone marrow stromal stem cells (BMSSCs), are multipotent stem cells capable of differentiating into various cell types, such as osteoblasts, odontoblasts, adipocytes, neural cells and so on, depending on the different inductive media [1]. STRO-1 has been identified on a variety of mesenchymal stem cells. In osteoblasts differentiation, DPSCs with proper culture medium could proliferate and differentiate into preosteoblasts, which still retain a self-renewing capability, and in osteoblasts, form a living autologous fibrous bone [2]. However, the medium for inducing odontoblasts differentiation which has been widely used has not been evaluated until now.

Matrix extracellular phosphoglycoprotein (MEPE), also called osteoblast/osteocyte factor 45 (OF45), as a novel bone-specific extracellular matrix protein, is expressed in hard tissue and related proteins [3]. The major function of MEPE is to regulate the differentiation and mineralization of hard tissue-related cells [4]. Moreover, its exact role as a regulator of mineralization in the differentiation of osteoblasts has been investigated. Several studies have demonstrated the promotive effects of MEPE on the mineralization of bone tissue [5,6], and the expression of MEPE is only observed in immature odontoblasts, and the MEPE becomes downregulated when the odontoblasts mature [7,8]. Recent studies have indicated that MEPE could exert a negative influence upon odontoblastic maturation [9]. These results agreed with recent results in laboratory studies of bone formation in MEPE-knockout mice, which have shown the increase of bone formation and bone mass [10]. In the research of the dentinogenesis, the information on the expression and regulation of MEPE in human dental pulp stem cells has not been widely investigated. Recently, Siggelkow *et al*. [11] used differing concentrations of β-GP to induce primary human osteoblasts and demonstrated that there is a progressive inhibition of MEPE gene expression when cultured in the presence of 5 mM β-GP, vitamin-C-phosphate and dexamethasone. However, no relevant research has been found on the phenomenon of differing concentrations of β-GP inducing the differentiation of DPSCs.

Dentin sialophosphoprotein (DSPP), a major non-collagenous matrix protein of odontoblasts, undergoes cleavage to DPP (dentin phosphoprotein) and DSP (dentin sialoprotein) [12]. DSP is associated with dentin mineralization [13] and is believed to play a crucial role in dentinogenesis. Previous reports have demonstrated that DSPP-null mice have shown tooth defects similar to human dentinogenesis imperfections in III-level patients with enlarged pulp chambers, increased width of predentin zone, hypomineralization, and pulp exposure [14]. Other reports have shown that DSP and DPP played distinct roles in dentin mineralization, DSP regulating initiation of dentin mineralization, and DPP being involved in the maturation of mineralized dentin [15].

Bone and dentin are all mineralized tissues, similar in their matrix protein composition as well, with much commonality between them [16]. Relevant report said that when DPSCs differentiated into osteogenic progenitors, they would change their antigen surface expression and morphology [17]. According to the osteoblasts, the mineralizing condition of differentiated odontoblasts is usually composed of 50 mg/mL vitamin-C-phosphate, 10 mM β-glycerophosphate (β-GP) and 10 nM dexamethasone [8,9,18]. Whereas a few researchers use 5 mM β-GP as an inductive concentration for odontogenic/osteogenic differentiation *in vitro*, they did not explain why they chose 5 mM β-GP in their study [19,20,21,22]. To the best to our knowledge, different concentrations of β-GP in mineralization solution have not yet been evaluated in odontoblast cultures.

One purpose of this study is to explore the influence of the differentiation DPSCs through the use of different concentrations of β-GP, in order to clarify the roles of β-GP in the differentiated process of inducing human dental pulp stem cells into odontoblasts. The other purpose is to ascertain the optimal mineralization culturing concentrations by means of detecting the expression of odontoblast-like marker DSP, to obtain a proper culture medium of odontoblasts for dental tissue engineering.

## 2. Results

### 2.1. Isolation and Collection of Primary Cultures

**Figure 1:**
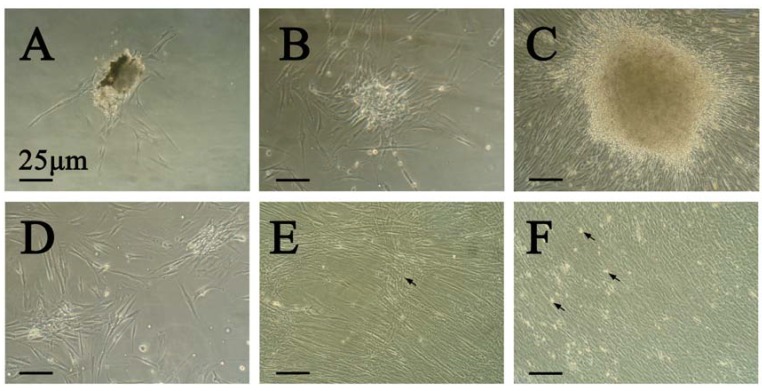
Morphological appearance of DPSCs. (original magnification, ×40). (**A**) The appearance of DPSCs at day 1 of culture. (**B**) DPSCs proliferate as a typical fibroblast-like morphology on day 7. (**C**) Cells started to form an aggregated center with increased extracellular matrix deposition. Generate a large clonogenic cell population after 2 week-culture. (**D**) Proliferation of DPSCs enters an active stage after passaging. (**E**) Formation of hill-and-valley organization (arrows nodules). (**F**) Cells retract and generate multicellular nodules (arrows nodules).

We cultured primary DPSCs, whose appearance is depicted in Figure 1. Cells adhered to the glass substratum on the 4th day (Figure 1A), and then generated clonogenic cell populations up to the 7th day which were characterized by a typical fibroblast-like morphology (Figure 1B). After 14 day-culture, the clonogenic cells were more homogenous, with typical fibroblastic shape and long cytoplasmic processes, tending to align themselves in parallel lines, and became large enough to passage (Figure 1C). Once passaged, the cells began to proliferate very quickly (Figure 1D). A long-term 28-day culture was chosen to continuously stimulate the third passage cells with a combination of FBS, different concentrations of β-GP, vitamin-C-phosphate, and Dex. Along with the progress of culture, cell retraction from other areas formed the distinct hill-and-valley-type of morphology (Figure 1E). A similar result has been reported by Proudfoot *et al.* [23]. After reaching confluence, the cells grew as monolayer, then created multiple layers and coalesced into multicellular foci or nodules (Figure 1F). Then the total protein was extracted at day 7, 14, 21, 28 respectively.

### 2.2. Characterization of the Immunophenotype and Differentiation Potential of DPSCs *in Vitro*

**Figure 2:**
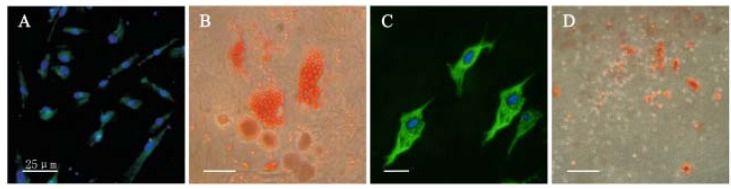
Multilineage differentiation capacity of DPSCs. (original magnification, ×40). (**A**) Cells are positive for STRO-1, staining by immunofluorescence. (**B**) Adipogenic differentiation was shown by the accumulation of neutral lipid vacuoles stainable with Oil Red O. (**C**) Immunofluorescence assay for nestin after 7 days’ indution of DPSCs. (**D**) Osteogenic differentiation was shown by the deposition of a mineralized matrix indicated by Alizarin Red S.

The cells expressed mesenchymal stem-cell markers STRO-1 were detected by immunofluorescent staining (Figure 2A). To assess the adipogenic potential, the cells were induced for 3 weeks. As shown in Figure 2, the formation of neutral lipid vacuoles was noticeable and visualized by Oil Red O staining. Meanwhile, after 7 days neuron induction of DPSCs, the cells in the flask acquired the morphology of neuron-like cells exhibiting a refractile cell body with extended neuron-like structures. Furthermore, the differentiated cells were positive for nestin (Figure 2C) and mineralized nodules were stained by Alizarin Red S. Figure 2D showed the successfully osteogenesis induction.

### 2.3. Cytotoxicity Assay

**Figure 3:**
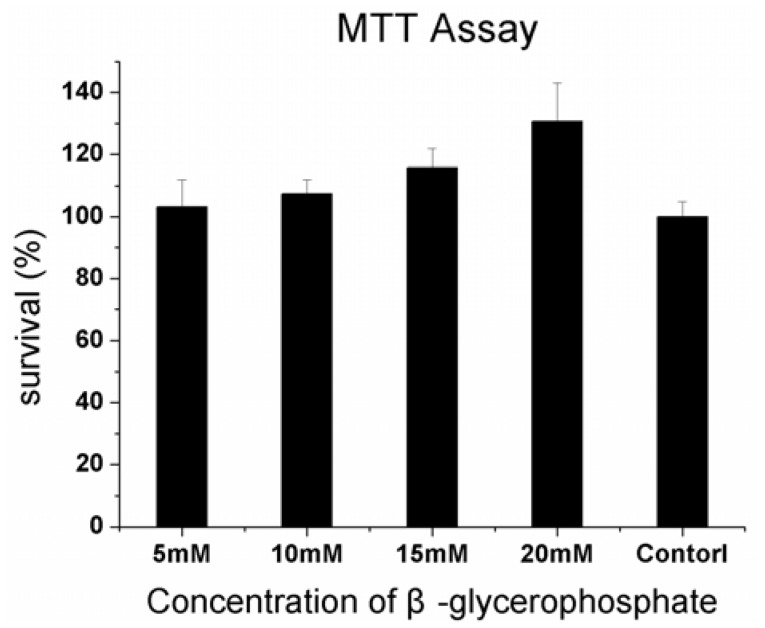
Cytotoxicity assay examined by MTT. Four concentrations of β-glycerophosphate did not inhibit the growth of DPSCs with the cell survival approximately 100%.

First, the effects of β-GP on cell viability were examined. As shown in Figure 3, β-GP showed no inhibition activity against DPSCs at concentrations ranging from 5–20 mM. This indicated that β-GP did not inhibit the growth of DPSCs. Otherwise, after treatment with 20 mM β-GP, cell survival increased to 130.68 ± 12.35%. Thus, it seems unlikely that the differentiation effects were due to any cytotoxic effects of β-GP.

### 2.4. Protein Expression in DPSCs Differentiation

**Figure 4:**
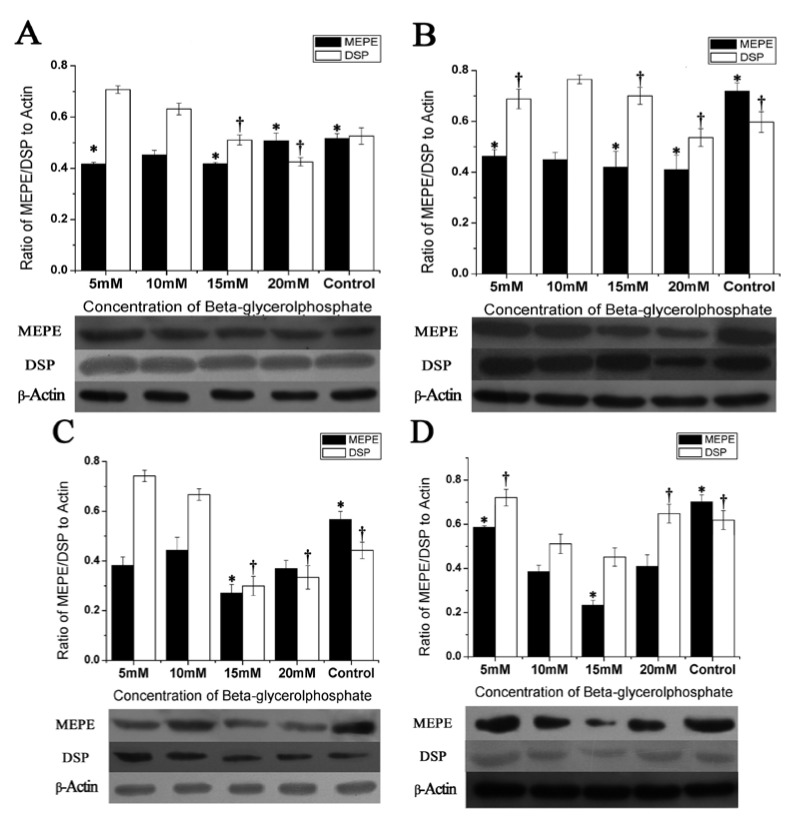
Western blot. Protein expression of MEPE and DSP in DPSCs culture after continuous incubation with different concentrations of β-glycerolphosphate. The relative protein expression was calculated by dividing the absolute level of expression of MEPE and DSP with the absolute level of expression of β-actin at 4 time points, at days 7 (**A**), 14 (**B**), 21 (**C**), 28 (**D**). Each point represents the value calculated from three different cell samples with the same cell sample in triplicate. * MEPE significant difference against 10 mM β-glycerolphosphate group, *p* < 0.05. ^†^ DSP significant difference against 10 mM β-glycerolphosphate group, *p* < 0.05.

In this project, we cautiously investigated the developmental and time-dependent protein expression of MEPE in comparison to the odontoblast characteristic protein DSP in DPSCs differentiation culture. We performed an experiment with different concentrations of β-GP at day 7, 14, 21 and 28 in culture to determine the relatively optimal conditions for differentiation of odontoblasts by detecting the expressions of MEPE and DSP, the results of which are summarized in Figure 4.

Protein detection showed that the expression of MEPE was opposite to that of DSP in all four mineralized groups in the first three weeks, while the expressions of MEPE and DSP showed the same tendency at the end of the culture. Noticeably the control group was very different from the mineralized groups with non-regular levels of MEPE and DSP expression, and the expression of MEPE was always the highest one throughout the whole period of the mineralization. However, at the end of the culture, the expression of DSP in control group turned to be higher than that in the first three weeks. During the whole culture period, except the last week, the tendency of MEPE expression was opposite to that of the DSP in all four mineralized groups. 

Taking a wide view of the whole period of mineralization, an apparently high expression of DSP with low levels of MEPE in 5 mM β-GP group could be seen throughout the whole culture period, with the sole exception of the 2nd week’s detecting. There was a small but significant high expression of DSP in 10 mM β-GP group compared with the 5mM β-GP group. In the meanwhile, 20 mM β-GP group was considered to be a poorly differentiated group with low expression of DSP in first three weeks but with an abnormal high level during the fourth week. However, an apparent inhibition of differentiation was revealed in 15 mM β-GP group since the 3rd week, the results of which have been summarized in Figure 4.

### 2.5. Mineralization Assay

**Figure 5:**
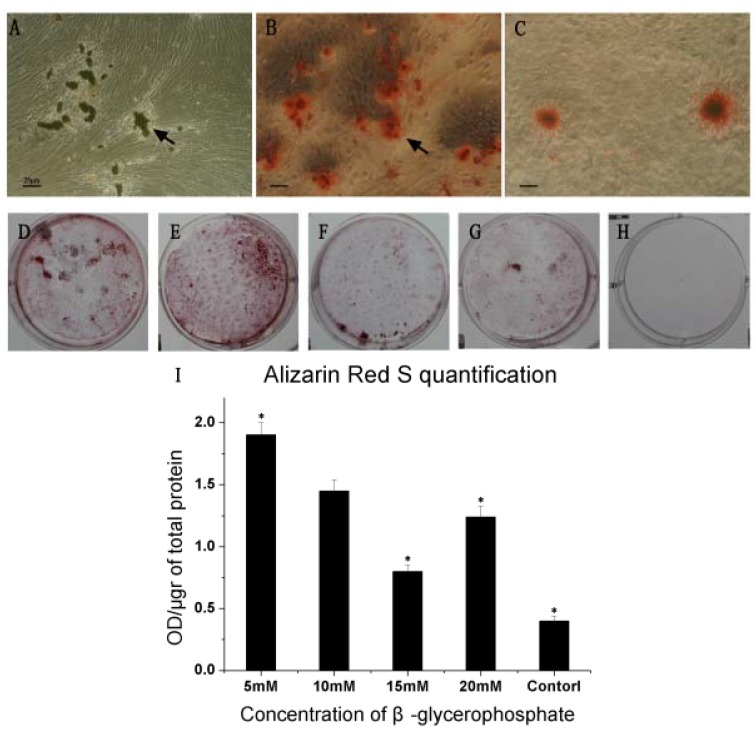
Cell morphology and mineralized nodules of human dental pulp cells maintained in mineralized medium for 28 days. (**A**) Arrow shows the cells started to form an aggregated center after the mineralized culture (original magnification, ×40). (**B**) Arrow shows the cells stained by Alizarin red S cultured in mineralized groups, (original magnification, ×40). (**C**) Cells stained by Alizarin red S cultured in control group (original magnification, ×40). (**D–H**) 5 groups of cells stained by Alizarin Red S shows that 5 mM β-glycerolphosphate group form more mineralized nodules. (**I**) Quantification of Alizarin Red S at 28th day. Data are shown as mean OD/µg of total protein ± SD (n = 6). * Indicates significant differences compared to10 mM group (*p* < 0.05).

After 14 days of cultivation, the first sign of calcification was found (Figure 5A); then, after 28 days, we detected the depositions of phosphate and calcium by Alizarin Red S. Microscopic examination revealed that, mineralization centers exhibited increased level of mineral deposition while the surrounding peripheral zone showed less advanced matrix mineralization (Figure 5B). In this study, both low- and high-concentration media initiated mineral deposition. The organized structures could also be observed in control group. However, the mineralization was very limited with few spontaneous nodules (Figure 5C). Figure 5D–H showed the mineralized formation in 5 groups. Quantification of mineralized nodule formation was represented as OD per μg of total cellular protein (Figure 5I). The data showed that 5 mM β-GP group had a prominent mineralized ability. 

## 3. Discussion

In this study, we investigated the developmental and time-dependent protein expression of MEPE in comparison with the odontoblasts’ characteristic protein DSP by means of inducing the differentiated process of transforming human dental pulp stem cells into odontoblasts, under differing concentrations of mineralization media. Western blot analyzed with an anti-MEPE antibody showed that only the control group synthesized and secreted the highest levels of MEPE protein, which proved that the mineralizing mediums have an apparently inhibitory effect on the MEPE protein expression, in order to advance the differentiation of the DPSCs. In the meanwhile, we found that the expressions MEPE *vs*. DSP tended to be inversely proportional, along with the process of DPSCs’ differentiating into odontoblasts, suggesting that the expression of MEPE related to DSP and can be used to monitor DPSC as they are used for studies of odontoblast differentiation, tissue engineering or vital pulp therapy [8]. Our experimental results showed that 5 mM β-glycerolphosphate group form more mineralized nodules than other groups. Therefore, we have reason to believe that 5 mM β-glycerolphosphate is more suitable than other concentrations in inducing the differentiation of odontoblast.

Dentinogenesis is a necessary prerequisite for dental tissue engineering. Human pulp cells can be induced *in vitro* to differentiate into cells of odontoblastic phenotype, characterized by polarized cell bodies and accumulation of mineralized nodules [24,25]. If seeded the colony cell onto dentin, some DPSCs convert into odontoblast-like cells with a polarized cell body and a cell process extending into the existing dentinal tubules [22,26]. Bone and dentin are mineralized tissues that closely resemble each other both in composition and in mechanism of formation. Furthermore, BMSSCs and DPSCs are not only able to differentiate into osteoblasts/odontoblasts *in vivo* early in the transplantation process, but also are capable of inducing host cells to participate in tissue regeneration by the formation of a hematopoietic marrow anda pulp-like complex. When DPSCs are seeded onto human dentin surfaces and implanted into immunocompromised mice, reparative dentin-like structure is deposited on the dentin surface [27]. Report on dentinogenesis modeled the molecular events in dental injury and reparative dentinogenesis by treating DPSC cells with a soluble extract of dentin, containing a cocktail of growth factors and bioactive molecules [28].

Sibling proteins, such as BSP, DMP-1, and DSPP all play positive roles in the process of odontoblast differentiation and maturation [12,27,29]. Current knowledge suggests that MEPE along with DSPP are potential odontogenetic differentiation markers [8]. DSP is considered to be a major marker of odontoblasts differentiation. It is widely used in the identification of odontoblasts [30,31,32,33]. However, the roles of MEPE as a regulator of mineralization are distinctly different in bone and in dentin. Several studies have revealed the promotive effects of MEPE upon the mineralization of bone tissue [5,6]. In contrast, mice with a targeted deletion of MEPE show increased bone formation and bone mass, clearly indicating that MEPE is an inhibitor of mineralization [10]. Relevant researches for MEPE demonstrate that there is a negative accommodation of MEPE as the odontoblasts differentiation [8]. MEPE down-regulates the differentiation of odontoblasts via its C-terminal fragment [9]. It is further observed that the expression of MEPE changes at different time points. Therefore, we cannot completely depend on the inducing condition of the osteoblasts despite the fact that the mineralization medium of osteoblasts works well in odontogenetic cultures.

This study is not the first one to discuss different culture mediums in inducing the differentiation of odontoblasts. In osteoblast differentiation, FBS at high percentage for a long time exerts a differentiation activity and, in particular, containing estrogens like the red phenol which is also in the medium favors an osteoblastic differentiation [34]. However, we are the first one to claim the effect of different concentrations of β-glycerolphosphate in the differentiation of DPSCs. Also, there is an influence of differing concentration of β-GP upon DPSC’s differentiation. As proper concentration of dexamethasone could stimulates the commitment of pulp cells to odontoblast-like cells, the proper concentration of β-GP would promote the maturation of DPSCs, while high concentrations of β-GP tend to inhibit the differentiation. The 10 mM concentration of β-GP has been wildly used as a component of mineralized medium. However, the level of inorganic phosphates is 1.0–1.5 mM in serum or plasma, whereas the organic phosphate level (which is mostly phosphor-lipid) is about 3 mM. However, the concentration of hydrolysable ester phosphate is 1.1 mM or less. Thus, a medium concentration of 10 mM β-GP is high when compared with physiological levels. The result of our experiment also confirmed that low concentrations of β-GP had a promotive effect on DPSCs’ differentiation with the expression of DSP increased. We thought that the DPSCs prefer to secrete DSP in the process of the mature and then form the extracellular matrix (ECM); MEPE in this process might not be required to express or be affected by other protein inhibitors. Thus, we found that the greater the cell matured, the lower the MEPE expressed, especially under the 5 mM β-GP concentration culture. This might be due to the process of DPSCs maturation, which accelerates the down-regulation of MEPE. At the end of the culture, the differentiated cells entered on their mature phase and the synthesization of MEPE could, at this point, not have influenced the differentiation. Apparently, MEPE accompanied with DSP expressed far higher than before, and the control group might avoid the inhibitory effect of MEPE and get into the state of differentiation climax. Alizarin Red S also proved that the control group generated mineralized nodules at 28 days detecting. In the meanwhile, lower expression of DSP at the last week’s detection in all four mineralized groups demonstrated that the cells had reverted to the terminal differentiation stage with poor ability to differentiate, even when the culture time was prolonged. It is possible that the cells created multiple layers and became too crowded to differentiate after the 28 day culture period. Additionally, we cannot exclude the possibility that an increase of MEPE expression could occur again beyond the time investigated. Furthermore, we have only measured the protein level both of MEPE and DSP and it is entirely possible that protein productions behave differently. Mineralization assay was performed to compare the differentiation capabilities between the different concentrations of β-GP culture conditions. All 5 groups succeeded in forming mineralized nodules, and 5 mM β-GP group formed more mineralized nodules by using the CPC extraction method. 

The down-regulation of the MEPE expression suggests that the differentiation of DPSCs is proceeding successfully, and the differentiation is coming into its prosperous stage when the gap between MEPE and DSP expression become bigger. It is interesting to investigate the relationship between MEPE and DSP in the differentiation of DPSCs induced by different concentrations of β-GP in the development of dental tissues. Meanwhile, in order to reach the high degree of mineralization, we can acquire the optimal inducing conditions by changing the β-GP concentration at different time points. In this manuscript, 5 mM β-GP seemed to be much better than 10 mM to induce the differentiation of DPSCs, leading to higher expression of DSP and lower expression of MEPE throughout the culture. Further studies on proteolytic processing for the activation of MEPE and the promotion of the DPSCs differentiation are in progress.

## 4. Experimental

### 4.1. Subjects and Cell Culture

Normal human third molars were collected from adults (19–29 years of age) at the Department of Oral and Maxillofacial Surgery in the 2nd Affiliated Hospital of Harbin Medical University; all the procedures were approved by the institutional ethics committee. Human DPSCs were isolated and cultured as previously reported [35], cultured at 37 °C, in 5% CO_2_ in DMEM F-12 with 15% fetal bovine serum (FBS, Hyclone, Logan, UT, USA), 100 units/mL penicillin and 100 mg/mL streptomycin (Beyotime, Shanghai, China). After 14 days of growth, DPSCs were detached using 0.25% trypsin/1mM EDTA (Beyotime).

### 4.2. Investigation of Stem Cell Marker by Immunofluorescent Staining

The cells grown on the coverslips were fixed in 4% paraformaldehyde for 15 min. After blocking with 1% complete serum for 2 h at 37 °C, STRO-1 (R&D systems, Minneapolis, MN, USA) was applied to incubate with the coverslips overnight at 4 °C. Image was captured after incubation of cells with FITC-conjugated (goat anti-mouse: Zymed, San Francisco, CA, USA) for STRO-1 assay for 30 min using the Olympus BX51 microscope (Tokyo, Japan).

### 4.3. Adipogenic, Neuron-like Cells and Osteogenic Differentiation

Cells were planted at a density of 3 × 10^3^ per cm^2^ for 3 weeks in culture medium supplemented with 0.5 µM isobutylmethylxanthine (IBMX), 50 µM indomethacin, and 0.5 µM dexamethasone. Adipogenic differentiation was detected by Oil Red O stain as an indicator of intracellular lipid accumulation. Neuron-like cells differentiation was induced by neural-inductive medium containing of 2% dimethylsulfoxide (DMSO), 200 µM butylated hydroxyanisode (BHA), 10 µM forskolin, 0.1 mM/L β-mercaptoethanol (β-ME) and 1 µM hydrocortisone for 4 days. Then, the expression of nestin (Chemicon, Temecula, CA, USA) was detected by immunofluorescent staining. Osteogenesis inductive medium was supplemented with 50 mg/mL vitamin-C-phosphate and 2 mM L-glutamine (GIBCO, Grand Island, NY, USA) and cultured for 21 days.

### 4.4. Cell Culture in β-GP-free or β-GP-containing Medium

We divided the flasks into four groups with five flasks per group. When cells reached 70% confluence, four concentrations of β-GP consisting of normal medium with 10 nM dexamethasone (Sigma, St. Louis, MO, USA) and 50 mg/mL vitamin-C-phosphate additives were used: 5 mM β-GP group; 10mM β-GP group; 15 mM β-GP group and 20 mM β-GP group. We used normal medium containing DMEM and 15% FBS as a control group. The medium was changed three times a week and changed with FBS-free for another 24-h culture before any analysis was taken.

### 4.5. Cytotoxicity Assay

Inhibition of cell proliferation of β-GP was measured by MTT assay [36]. Briefly, DPSCs were plated in 96-well culture plates (1 × 10^5^ cells/well) separately. After 24 h incubation, normal DPSCs were treated with β-GP (5, 10, 15 and 20 mM, eight wells per concentration) for 28 days. MTT solution (5 mg/mL) was then added to each well. After 4 h incubation, the formazan precipitate was dissolved in 100 µL dimethyl sulfoxide, and then the absorbance was measured in an ELISA reader (Thermo Molecular Devices Co., Union City, CA, USA) at 570 nm. The cell viability ratio was calculated by the following formula: 

Inhibitory ratio (%) = [(OD_control_ − OD_treated_)/(OD_control_)] × 100%

### 4.6. Western Blot Analysis

For isolation of total protein fractions, cells were collected, washed twice with ice-cold PBS, and lysed using cell lysis buffer [20 mM Tris pH 7.5, 150 mM NaCl, 1% Triton X-100, 2.5 mM sodium pyrophosphate, 1 mM EDTA, 1% Na_3_CO_4_, 0.5 μg/mL leupeptin, 1 mM phenylmethanesulfonyl fluoride (PMSF)]. The lysates were collected by scraping from the plates and then centrifuged at 10,000 rpm at 4 °C for 5 min. Total protein samples (20 μg) were loaded on a 12 % of SDS-polyacrylamide gel for electrophoresis, and transferred onto PVDF transfer membranes (Millipore, Billerica, Bedford, MA, USA) at 0.2 μm for 1.5 h. Membranes were blocked at room temperature for 2 h with blocking solution (1% BSA in PBS plus 0.05% Tween-20). Membranes were incubated overnight at 4 °C with the following antibodies: DSP (1:500; Santa Cruz, CA, USA), MEPE (1 μg/mL; R&D Systems, Minneapolis, MN, USA) and β-actin (1:500, Zhongshan Goldenbridge, Beijing, China). Secondary antibodies were peroxidase-conjugated goat anti-mouse IgG (H+L), and rabbit anti-goat IgG (1:10,000, Zhongshan Goldenbridge, Beijing, China). Immunoblot bands were visualized by enhanced chemiluminescence with the ECL Western blot kit (CWBIO, Beijing, China) and Tanon Gis-1000 digital image gel analytical system (Shanghai Tanon Science Company, Shanghai, China) was used for photography and protein-band assay.

### 4.7. Mineralization Induction and Quantification

The third passage cells were seeded into 6-well plates (Costar) at an initial density of 5 × 10^4^ cells/well. Five wells were cultured with or without four concentrations of mineralization medium for 28 days without passaging, but replacing the medium twice a week, after which calcium accumulation was detected by fixing the cultures with 95% ethanol for 30 min and then staining with 0.1% Alizarin Red S (Sigma) in 37 °C for 30 min. To quantify matrix mineralization, the alizarin red S stained cultures were incubated with 100 mM cetylpyridinium chloride for 1 h at 37 °C to solubilize and release calcium-bound alizarin red into solution. Subsequently, 200 mL aliquots were transferred to a 96-well plate and the OD_570 nm_ of the solution was measured using a microplate reader (Shanghai Tanon Science Company). Mineralized nodule formation was represented as OD per μg of total cellular protein, determined by Bradford Protein assay. Experiments were performed in triplicate wells and repeated at least three times.

### 4.8. Statistical Analysis

All values are expressed as mean ± standard error of the mean (SEM). Statistical analysis was performed by using one-way analysis of variance (ANOVA) and then Bonferroni multiple comparisons (SPSS Software; SPSS Inc, Chicago, IL, USA). Statistical significance was accepted at *p* < 0.05.

## 5. Conclusions

In conclusion, mineralized groups obviously inhibited the expression of MEPE, especially for the 5 mM β-glycerolphosphate group. Therefore, the routine culture system (10 mM β-glycerolphosphate) is not as good as 5 mM to down-regulate the expression of MEPE. We should change the concentration of β-glycerolphosphate to maintain the phenotype of odontoblasts and get better differentiation for dentinogenesis. Low expression of MEPE and high level of DSP could identify the successful differentiation of DPSCs. It is therefore reasonable to insist that MEPE accompanied with DSP should be considered as a marker of DPSCs differentiation.
